# Dissecting the chromosomal composition of mutagen-induced micronuclei in *Brachypodium distachyon* using multicolour FISH

**DOI:** 10.1093/aob/mcy115

**Published:** 2018-07-05

**Authors:** Arita Kus, Jolanta Kwasniewska, Joanna Szymanowska-Pułka, Robert Hasterok

**Affiliations:** 1 Department of Plant Anatomy and Cytology, Faculty of Biology and Environmental Protection, University of Silesia in Katowice, Katowice, Poland; 2 Department of Biophysics and Plant Morphogenesis, Faculty of Biology and Environmental Protection, University of Silesia in Katowice, Katowice, Poland

**Keywords:** BAC clones, *Brachypodium distachyon*, chromosome-specific markers, induced genome instability, maleic hydrazide, mcFISH, micronuclei, molecular cytogenetics, mutagenesis, mutagens, X-rays

## Abstract

**Background and Aims:**

*Brachypodium distachyon* (Brachypodium) is a model species for temperate cereals and other economically important grasses. Its favourable cytogenetic features and advanced molecular infrastructure make it a good model for understanding the mechanisms of instability of plant genomes after mutagenic treatment. The aim of this study was to qualitatively and quantitatively assess the composition and origin of micronuclei arising from genomic fracture, and to detect possible ‘hot spots’ for mutagen-induced DNA breaks.

**Methods:**

Seeds of Brachypodium were treated with maleic hydrazide (MH) or X-rays. The structure of mutagen-induced micronuclei was analysed in root-tip meristematic cells using multicolour fluorescence *in situ* hybridization (mcFISH) with various repetitive (5S rDNA, 25S rDNA, telomeric, centromeric) and low-repeat [small and large pools of bacterial artificial chromosome (BAC) clones specific for chromosome Bd1] DNA sequences.

**Key Results:**

The majority of micronuclei derive from large, acentric fragments. X-rays caused more interstitial DNA breaks than MH. Double-strand breaks rarely occurred in distal chromosome regions. Bd1 contributed to the formation of more mutagen-induced micronuclei than expected from random chromosome involvement.

**Conclusions:**

mcFISH with chromosome-specific BAC clones offers insight into micronuclei composition, in so far as it allows their origin and formation to be determined more specifically. A reliable assay for micronuclei composition is crucial for the development of modern genotoxicity tests using plant cells. The combination of mutagenic treatments and well-developed cytomolecular resources in Brachypodium make this model species very promising for plant mutagenesis research.

## INTRODUCTION

There are numerous chemical and physical agents that can affect the structure of DNA and cause double-strand breaks (DSBs), which may result in chromosome aberrations and formation of micronuclei (MN), eventually leading to DNA loss. Physical agents such as X-rays can damage DNA throughout the cell cycle, inducing the formation of apurinic/apyrimidinic sites, pyrimidine dimers and DNA–DNA cross-links. In contrast, some chemical mutagens, including maleic hydrazide (MH), are S-phase-dependent, although the exact mechanism of action of this mutagen is not fully understood and some of the effects that it imposes cannot be correlated with S-phase. MH seems to affect the biosynthetic activity of the nucleolus and may also act as an inhibitor of the synthesis of nucleic acids and enzymes that are involved in the mitotic spindle, causing multipolar anaphases, lagging chromosomes and chromosome breaks (Marcano *et al.*, 2004). Many studies on mutagen genotoxicity are now based on the scoring of micronuclei in plants (Vaijapurkar *et al.*, 2001; Misik *et al.*, 2016) and mammals (Avlasevich *et al.*, 2006; Lau *et al.*, 2009; Hashimoto *et al.*, 2010). First proposed by Evans *et al.* (1959), the MN test is a standard assay, which is widely used to screen for potential genotoxic agents *in vitro* and *in vivo*. The MN test has so far been successfully applied in several monocots, such as *Allium sativum* (Unyayar *et al.*, 2006), *Allium cepa* (Leme and Marin-Morales, 2008; Pesnya and Romanovsky, 2013), *Triticum aestivum* (Li *et al.*, 2008), barley (Jovtchev *et al.*, 2010), *Brachypodium distachyon* (Brachypodium) (Kus *et al.*, 2017) and dicots, for example *Vicia faba* (Minouflet *et al.*, 2005; Khadra *et al.*, 2012). Examination of large chromosome aberrations is feasible using simple classical cytological methods such as the Feulgen reaction. However, molecular cytogenetic approaches, especially fluorescence *in situ* hybridization (FISH), are much more effective in detecting a variety of small chromosome rearrangements. Furthermore, the application of a micronucleus test combined with FISH can identify specific chromosomes or chromosome fragments in micronuclei. Until recently, the dearth of FISH probes that can specifically target individual chromosomes in plants has meant that the identification of chromosome aberrations was based entirely upon the use of repetitive DNA, such as centromeric, telomeric and ribosomal DNA (rDNA) sequences as probes for FISH (Siroky *et al.*, 2003; Rocha *et al.*, 2016). Such probes have been useful in determining the origin of micronuclei in barley (Jovtchev *et al.*, 2002; Juchimiuk *et al.*, 2007; Juchimiuk-Kwasniewska *et al.*, 2011), *Crepis capillaris* (Maluszynska *et al.*, 2003) and Brachypodium (Kus *et al.*, 2017). The last of these studies exploited the variety of favourable cytological features of this plant, and its relatively high sensitivity to mutagens, demonstrating that Brachypodium can also serve as a useful model organism in plant mutagenesis studies.

Chromosome painting (CP), which permits the precise and selective visualization of entire chromosomes or their specific segments both during cell division and interphase, is one of the most advanced FISH-based approaches (Tucker, 2015). To detect the chromosomal rearrangements that are connected with particular diseases in humans, painting chromosomes had been used widely for some time (Schrock *et al.*, 1996; Adam *et al.*, 2006; Cooper *et al.*, 2006; Dufke *et al.*, 2006; Guo *et al.*, 2012). CP has also been applied successfully to determine the involvement of specific chromosomes in the formation of micronuclei after *in vitro* exposure of human cells to chemicals (Guttenbach and Schmid, 1994; Fauth *et al.*, 2000; Chung *et al.*, 2002; Hovhannisyan *et al.*, 2012) and radiation (Fimognari *et al.*, 1997; Braselmann *et al.*, 2003). Some of these studies have indicated that micronuclei preferentially comprise particular chromosomes (Fimognari *et al.*, 1997; Leach and Jackson-Cook, 2001; Chung *et al.*, 2002; Norppa and Falck, 2003), which may be related to chromosome size, gene density (Puerto *et al.*, 1999) and other aspects of the chromatin organization (Fauth *et al.*, 2000).

In contrast to animals and humans, CP in plants is currently limited to several small-genome genera, such as *Arabidopsis* and some of its close relatives in Brassicaceae (Lysak *et al.*, 2006), *Brachypodium* (Idziak *et al.*, 2011), *Solanum* (Szinay *et al.*, 2012) and *Cucumis* (Lou *et al.*, 2014). CP has been used in the genus *Brachypodium* to study chromosome and karyotype structure and evolution (Betekhtin *et al.*, 2014; Idziak *et al.*, 2014), as well as to track the arrangement of chromosome territories in interphase nuclei (Robaszkiewicz *et al.*, 2016). To the best of our knowledge, CP has not yet been applied to investigate mechanisms of chromosomal aberrations in plants.

In this study, we used multicolour FISH (mcFISH) and CP to characterize the composition and to decipher the origin of micronuclei in Brachypodium root-tip meristematic cells resulting from the application of chemical and physical mutagens. We show that this approach permits effective qualitative and quantitative analyses of micronuclei, and detects putative ‘hot spots’ of DNA breaks. We demonstrate that CP significantly improves the sensitivity of the ‘standard’ MN test, which is commonly used for genotoxicity tests in plant cells.

## MATERIALS AND METHODS

### Plant material and mutagenic treatment

Seeds of Brachypodium (2*n* = 10) reference genotype Bd21 were sourced from the collection held by the United States Department of Agriculture – National Plant Germplasm System. MH (Sigma-Aldrich) at concentrations of 3 and 4 mm and X-radiation at doses of 125 and 150 Gy were used for the mutagenic treatment. The MH concentrations used here are known to induce micronuclei in Brachypodium cells (Kus *et al.*, 2017). Before chemical treatment, the seeds were presoaked in distilled water for 6 h and then treated with MH for 3 h followed by germination in Petri dishes on moistened filter paper for 3 d at 21 °C in the dark. Irradiation was carried out at the Silesian Centre for Environmental Radioactivity, Central Mining Institute in Katowice, Poland, at a dose rate of 578.4 mGy min^–1^ using an XCS-320-ST/X-RAY-CAL X-ray machine (TEMA) equipped with a 320-kV ceramic lamp. Irradiations were performed at 20.3 ± 0.1 °C, under a pressure of 985 hPa and 27–33 % relative humidity. After irradiation, the seeds were germinated as described above. Whole seedlings were fixed in a mixture of 3:1 (v/v) methanol/glacial acetic acid and stored at −20 °C until use. The MH treatment was repeated three times.

### Cytogenetic preparation

Cytogenetic preparations of root meristems were done as described by Jenkins and Hasterok (2007). Excised root tips were digested for 1.5 h at 37 °C in a mixture of enzymes comprising 6 % (v/v) pectinase (Sigma-Aldrich), 1 % (w/v) cellulase (Sigma-Aldrich) and 1 % (w/v) cellulase ‘Onozuka R-10’ (Serva).

### Probes, labelling and mcFISH

5S rDNA, telomeric, centromeric and 25S rDNA (which detects the sites of 35S rDNA) probes were used in the first FISH experiment. Their origin, composition and labelling are as described in Kus *et al.* (2017), except that the telomeric and centromeric probe were labelled with tetramethylrhodamine-5-dUTP and digoxigenin-11-dUTP (both Roche Diagnostics), respectively.

In the second FISH experiment, small pools containing five low-repeat bacterial artificial chromosome (BAC) clones (Supplementary Data Table S1) were selected to paint the subterminal regions of chromosome Bd1. To paint the entire arms of Bd1, large pools of 23–24 BACs were assembled for the third FISH experiment (Table S2). All clones were derived from the BD_ABa and BD_CBa Brachypodium genomic DNA libraries and were selected from the FingerPrinted Contigs that had previously been assigned to chromosome Bd1 (IBI, 2010). To avoid unwanted cross-hybridization, only low-repeat BACs (i.e. containing < 40 % of repetitive sequences) were selected (Tables S1 and S2) using RepeatMasker (http://www.repeatmasker.org;Febrer *et al.*, 2010). The clones comprising a given pool were bulk-isolated using standard alkaline extraction as described previously (Farrar and Donnison, 2007). Individual pools were labelled by nick-translation with either digoxigenin-11-dUTP or biotin-16-dUTP, or with digoxigenin-11-dUTP and tetramethylrhodamine-5-dUTP in equal proportions (Hasterok *et al.*, 2002). The telomeric probe HT100.3 was labelled as in the first experiment.

FISH was performed according to Jenkins and Hasterok (2007) with minor modifications. The hybridization mixture consisted of 50 % deionized formamide, 10 % dextran sulphate, 2× saline sodium citrate, 0.5 % sodium dodecylsulphate and 2.5–3.0 ng mL^–1^ of each DNA probe. Post-hybridization washes were equivalent to about 60 % stringency. Digoxigenated probes were immunodetected using FITC-conjugated anti-digoxigenin antibodies (Roche Diagnostics), while the biotinylated probes were detected using Alexa Fluor 647-conjugated anti-biotin antibodies (Jackson Immuno Research). The preparations were mounted and counterstained as in Kus *et al.* (2017).

### Image acquisition and processing

All photomicrographs were acquired using an Axio Imager.Z.2 (Zeiss) wide-field fluorescence microscope equipped with an AxioCam Mrm (Zeiss) high-sensitivity monochromatic camera, digitally coloured using Wasabi (Hamamatsu Photonics), and (if required) uniformly processed to improve contrast and brightness and superimposed using Photoshop CS3 (Adobe). The total numbers of DAPI-stained nuclei with micronuclei in 2000 interphase cells in both the control and the treated material were recorded. In the FISH experiments, the numbers of micronuclei with and without specific signals were counted. For each experimental group, a total of 300 nuclei with micronuclei on three individual slides, each made from one meristem, were evaluated.

### Statistical analyses

Statistical analysis was performed where a non-zero signal was observed. Based on our previous observations, we assumed a normal distribution of the frequencies of nuclei with micronuclei. For this reason, parametric ANOVA tests followed by a post-hoc least significant difference (LSD) test were applied to detect any statistical differences between the frequencies of specific types of nuclei with micronuclei. Detailed statistical analyses relating to the data that are presented in the respective figures are provided in Table S3.

## RESULTS AND DISCUSSION

### X-radiation promotes micronuclei formation in Brachypodium cells

In the present study, X-rays were used for the first time as a mutagen to induce micronuclei in Brachypodium root-tip meristematic cells. Using DAPI staining, we estimated the frequencies of cells with micronuclei after seed irradiation with 125 or 150 Gy of X-rays and observed an increase in the frequencies of nuclei with micronuclei compared to the control.

The results permitted the clastogenic effects of X-rays to be compared with those that are induced in Brachypodium cells by other mutagenic agents. The frequency of cells with micronuclei that were induced by X-rays ranged from from 6.1 % to 6.3 % and was higher than the frequency of 5.6 % induced by 4 mm MH (Kus *et al.*, 2017). This difference may be because X-radiation acts during the entire cell cycle (Evans and Scott, 1964), while MH acts only during S-phase (Michaelis and Rieger, 1968). The results presented here confirm and extend our previous findings regarding the sensitivity of Brachypodium cells to mutagens and demonstrate its usefulness as a model monocot plant in mutagenesis studies.

### Micronuclei often originate from acentric fragments

**Fig. 1.**
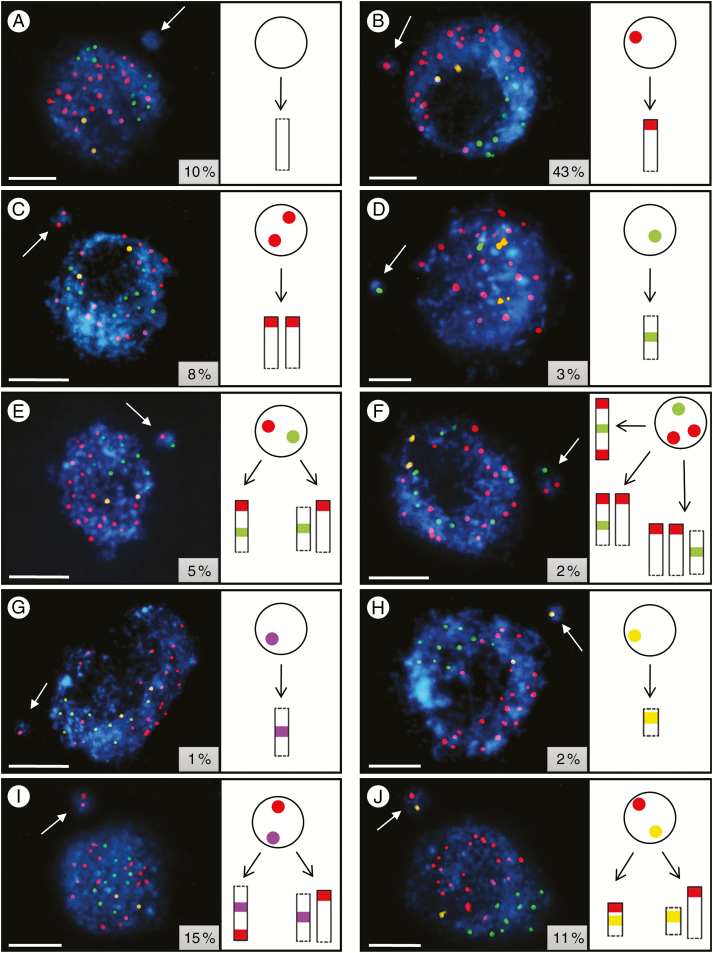
Brachypodium interphase nuclei with micronuclei induced by X-radiation and subjected to mcFISH with telomeric (red), centromeric (green), 5S rDNA (purple) and 25S rDNA (yellow) probes. The datasets for two doses of X-rays (125 and 150 Gy) have been combined. (A–J) Ten different kinds of micronuclei can be distinguished: (A) without any FISH signals, (B) with one telomeric signal, (C) with two telomeric signals, (D) with one centromeric signal, (E) with one centromeric and one telomeric signal, (F) with one centromeric and two telomeric signals, (G) with one 5S rDNA signal, (H) with one 25S rDNA signal, (I) with one 5S rDNA and one telomeric signal, and (J) with one 25S rDNA and one telomeric signal. Chromatin is stained with DAPI (blue). White arrows indicate the micronuclei and the insets show their frequencies. Diagrams next to the photomicrographs show the putative origins of the micronuclei. Transverse dashed lines indicate chromosome breakpoints. Scale bars = 5 µm.

Kus *et al.* (2017) simultaneously hybridized to chromosomes and interphase nuclei of control and MH-treated material with 5S rDNA, 25S rDNA, telomeric and centromeric probes (Fig. S1). In this study, we used 5S rDNA and 25S rDNA probes to analyse the involvement of chromosomes Bd4 and Bd5 in the formation of micronuclei in material that was subjected to X-radiation. The use of centromeric and telomeric probes allowed us to discern whether entire chromosomes or only their fragments form micronuclei. A quantitative analysis based on the presence or absence of particular FISH signals revealed ten types of X-ray-induced micronuclei, examples of which are shown in Fig. 1. Moreover, the application of mcFISH permitted the frequencies of micronuclei with different repetitive DNA signal composition to be determined. Because the frequencies of micronuclei with specific types of FISH signals did not depend on the doses of X-ray that were applied, all of the data were combined into one dataset.

Micronuclei without FISH signals (Fig. 1A) comprise interstitial, acentric part(s) of unidentified chromosome(s), and are observed with a relatively high frequency of 10 %. In contrast, after MH treatment only 5 % of micronuclei were FISH-negative (Kus *et al.*, 2017), suggesting that X-rays may cause interstitial DNA breaks in Brachypodium chromosomes at a higher frequency compared to MH. Surprisingly, in barley FISH-negative micronuclei that had been induced by gamma rays, MH and *N*-nitroso-*N*-methylurea treatment were observed with a similar frequency of 15 % (Juchimiuk *et al.*, 2007; Juchimiuk-Kwasniewska *et al.*, 2011). The presence of only one telomeric signal (Fig. 1B) in a micronucleus suggests the involvement of an acentric chromosome fragment. Quantitative analysis revealed that 43 % of all of the micronuclei have one telomeric signal only, which is comparable with the 45 % reported in our previous study with MH (Kus *et al.*, 2017). In X-ray-treated Brachypodium cells, micronuclei containing two telomeric signals are observed at a frequency of 8 %, which may be derived from two acentric fragments of unknown chromosomes (Fig. 1C). We also observe micronuclei that have a single centromeric signal (Fig. 1D), which implies the involvement of an interstitial region of an unidentified chromosome. Only 3 % of this type of micronuclei are observed in Brachypodium after X-ray treatment, which may be related to the fact that the formation of a micronucleus containing only a centromeric signal requires two DSBs, while only one is required to form a micronucleus with one telomeric signal. Interestingly, no micronuclei with only a centromere-specific signal were observed in barley after the application of gamma rays (Juchimiuk-Kwasniewska *et al.*, 2011). These authors indicate that large interstitial fragments that include only centromeric DNA are not generated by physical treatment in barley, but are formed by MH treatment. Our results clearly indicate that micronuclei in Brachypodium cells after X-ray treatment usually arise from the distal, acentric parts of chromosomes. This is in agreement with the findings of Kunzel *et al.* (2001), who reported that small, distal chromosome regions are the preferred sites for translocation breakpoints that are induced by gamma radiation in barley. The application of centromeric and CP probes in human leukocytes showed that mitomycin C-induced micronuclei comprise both chromosome fragments and whole chromosomes, and are predominantly composed of acentric fragments (Hovhannisyan *et al.*, 2012). The same observations were made by Lindberg *et al.* (2008) in human lymphocyte micronuclei.

The presence of one telomeric signal and one centromeric signal (Fig. 1E) may indicate a more complex origin, either from one chromosome arm containing the centromere or from two fragments: one interstitial and centric and the other acentric. Only 5 % of the micronuclei are characterized by the presence of both telomeric and centromeric DNA sequences. This type of micronucleus was observed at a similar frequency (7 %) after MH treatment in Brachypodium (Kus *et al.*, 2017). An even more complex constitution is manifested by 2 % of the micronuclei with one centromeric and two telomeric signals (Fig. 1F), because there are three possibilities for their origin. One is from a laggard Bd1, Bd2 or Bd3 chromosome, but an origin from two (one chromosome arm containing the centromere and one acentric fragment) or even three (one interstitial centric and two acentric) fragments of unidentified chromosomes also cannot be ruled out.

The micronucleus in Fig. 1G has a 5S rDNA signal, which clearly suggests its origin from an interstitial fragment of the long arm of chromosome Bd4 that involves the rDNA locus. The 35S rDNA-positive type of micronuclei (Fig. 1H) suggests that the interstitial fragment of the short arm of chromosome Bd5 is involved in their formation. These two types of micronuclei are observed at low frequency (1 and 2 %, respectively) after X-rays. Interestingly, similar frequencies were reported in Brachypodium cells after MH treatment (Kus *et al.*, 2017).

We also observed micronuclei that had both 5S rDNA and telomeric signals (Fig. 1I), which may indicate their formation from an acentric fragment of the long arm of chromosome Bd4 that involves 5S rDNA, or from two fragments – one acentric and one having the 5S rDNA locus. Micronuclei with these types of signals were relatively frequent (15 %). By contrast, 11 % of the micronuclei had one telomeric signal and one 25S rDNA signal (Fig. 1J). The presence of one 25S rDNA and one telomeric signal in a micronucleus implies its origin from an acentric fragment of the short arm of chromosome Bd5 that involves 35S rDNA, or from the interstitial fragment of chromosome Bd5 that carries these genes and the terminal fragment of an unidentified chromosome. Our results suggest that chromosome Bd4 is involved in X-ray-induced micronuclei formation more often than chromosome Bd5. Similar differences in the involvement of the 5S rDNA- and 35S rDNA-bearing chromosomes in micronuclei formation were observed in Brachypodium cells that had been subjected to MH (Kus *et al.*, 2017), and in barley after gamma ray treatment in spite of the fact that the genome of the latter species has four different chromosomes that have 5S rDNA loci and two other chromosomes that contain 35S rDNA loci (Juchimiuk-Kwasniewska *et al.*, 2011). The participation of specific chromosomes of *A. thaliana* in the formation of anaphase bridges was also demonstrated by FISH with BAC clones combined with a 25S rDNA probe (Siroky *et al.*, 2003). It was observed that 35S rDNA regions located at the ends of chromosomes 2 and 4 were involved in chromosome bridge formation at a higher frequency than might be expected from random fusion events.

To conclude, simultaneous and multicolour probing with four repetitive DNA sequences that are routinely used in FISH-based analyses of plant genomes is effective in the initial analysis of the involvement of specific DNA sequences or chromosome regions in micronuclei formation in Brachypodium. The relative frequencies of the different types of X-ray-induced micronuclei are consistent with those of chemical mutagen-induced micronuclei (Kus *et al.*, 2017).

### Large, distal chromosome fragments usually contribute to micronuclei formation

**Fig. 2.**
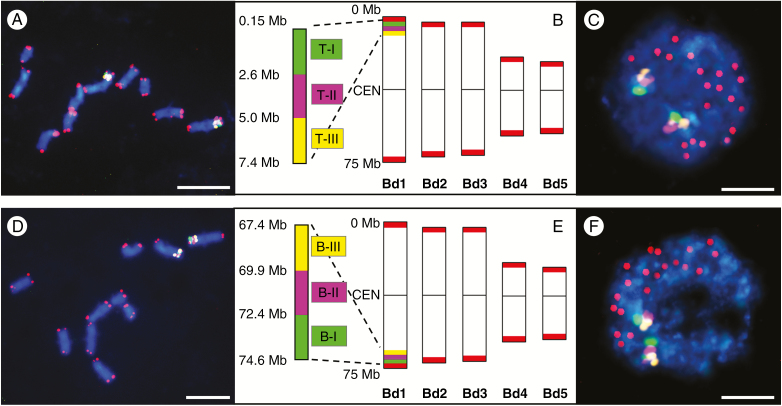
mcFISH with the small Bd1-specific BAC pools and the telomeric probe (red) in the control material. (A–C) The T-I (green), T-II (purple) and T-III (yellow) pools span the subterminal region of Bd1T. (D–F) The B-I (green), B-II (purple) and B-III (yellow) pools span the subterminal region of Bd1B. (A, D) Mitotic metaphase chromosomes. (B, E) Ideograms of Brachypodium chromosomes with the approximate physical positions of the pools. CEN, centromere. (C, F) Interphase nuclei without micronuclei. Chromatin is stained with DAPI (blue). Scale bars = 5 μm.

To discern the chromosome-specific origin of the MH- and X-ray-induced micronuclei, we selected pools of low-repeat BAC-based probes in combination with the telomeric probe that marks the physical ends of chromosomes. In this study, we chose Bd1 as the model chromosome due to the large number of specific BAC clones that are available for its contiguous painting (Idziak *et al.*, 2011). Another important consideration is that Bd1 is the longest chromosome in the complement and is almost metacentric. These features make it more feasible to assess and quantify the involvement of its top (Bd1T) and bottom (Bd1B) arms in micronuclei formation. To discriminate the different parts of the subterminal regions of Bd1 that are prone to form micronuclei, we set up three small and differentially labelled BAC pools, T-I (green), T-II (purple) and T-III (yellow) that were specific to Bd1T, and three other small pools (B-I, B-II and B-III; the same colour coding) that hybridize to Bd1B. We first visualized them in the control (i.e. non-irradiated) material and obtained a satisfactory mapping resolution (Fig. 2). These pools were separated from each other on the physical map (Table S1) and spanned approx. 7.4 Mb of its total length (Fig. 2B). In mitotic metaphase chromosomes, they give continuous painting signals in the distal part of Bd1T. Although these signals partially overlapped due to the highly condensed chromatin, they were clearly proximal to the telomeric probe (red) (Fig. 2A). A similar arrangement was observed for the BAC B-I, B-II and B-III pools, which spanned approximately 7.3 Mb of Bd1B (Fig. 2D, E). When hybridized to interphase nuclei, all of the pools are visualized at significantly higher mapping resolution, and their almost separate signals were accompanied by 20 signals of the telomeric probe (Fig. 2C, F).

**Fig. 3.**
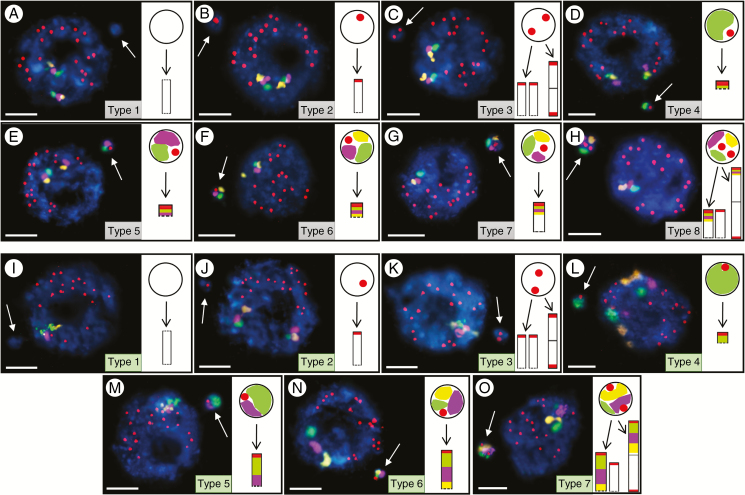
Brachypodium interphase nuclei with micronuclei induced by MH treatment and X-radiation that were subjected to mcFISH with the same set of probes as in Fig. 2A–C (A–H, small BAC pool) and Fig. 5A–C (I–O, large BAC pool). Various types of micronuclei are distinguished and their detailed composition is described in the main text. Chromatin is stained with DAPI (blue), and white arrows indicate the micronuclei. Diagrams next to the photomicrographs show the putative origins of the micronuclei. Transverse dashed lines indicate chromosome breakpoints. Scale bars = 5 µm.

After mutagenic treatment, we observe eight categories of micronuclei that either have no FISH signals or have signals in different combinations. The same types of micronuclei appear in material that was subjected to MH or X-rays. Because the same types of micronuclei were observed for both the top and the bottom arms of Bd1, only the nuclei with micronuclei with the telomeric probe and T-I, T-II and T-III pools are presented in Fig. 3A–H. The photomicrographs of micronuclei with telomeric probe and BAC pools spanning Bd1B are shown in Fig. S2. Although the FISH-negative micronuclei (Fig. 3A; type 1) were composed of an interstitial chromosome fragment of unknown origin, it does not exclude the possibility that the proximal part of chromosome Bd1 (not covered by the BACs) is involved. As in our previous FISH experiment, we observed micronuclei with both one (Fig. 3B; type 2) and two (Fig. 3C; type 3) telomeric signals, which implies that either one or two distal chromosome fragments were involved in their formation. Micronucleus type 4 was characterized by the presence of one telomeric signal and one signal that was derived from the T-I pool probe (Fig. 3D). Because two copies of the tri-coloured (green–purple–yellow) BAC-FISH painting signal were present in the nucleus with only 19 telomeric signals, this type of micronucleus may arise from a single DSB that occurred within the T-I pool. The presence of one telomeric signal and the BAC signal encompassing the T-I and T-II pools defined the content of micronucleus type 5 (Fig. 3E). Because this observation correlates with the presence of one tri-coloured painting signal in the original nucleus and an additional one, which was missing the part (green) that was diagnostic for the T-I pool, it seems likely that this micronucleus type results from a single DSB within the chromosome region covered by the T-II pool. Micronucleus type 6 (Fig. 3F) is composed almost exclusively of four signals, one from the telomeric probe and three others related to the BAC T-I, T-II and T-III pools. Because one of the yellow signals that is present in the original nucleus is not accompanied by any other signals, the logical conclusion is that a single DSB has taken place within the chromatin region that is marked by pool T-III. The organization of micronucleus type 7 is similar to one described previously, except that a higher proportion of FISH-negative chromatin is present (Fig. 3G). This, coupled with the presence of only one copy of the tri-coloured BAC-FISH signal in the original nucleus, suggests that the DSB causing this particular type of micronucleus occurs in the FISH-negative region of Bd1 located proximally to the chromosome region marked by the BAC T-I, T-II and T-III pools. Finally, we also observed micronucleus type 8, which contains two telomeric signals and three BAC signals, each of the latter of which is related to a different pool (Fig. 3H). Given that the original nucleus contains only one copy of the tri-coloured painting signal, this could imply that one complete, laggard chromosome Bd1 is involved in the formation of this type of micronucleus. Alternatively, such a micronucleus could be composed of two fragments: one consisting of the arm of Bd1 that carries the BAC-FISH landmarks and one distal fragment.

**Fig. 4.**
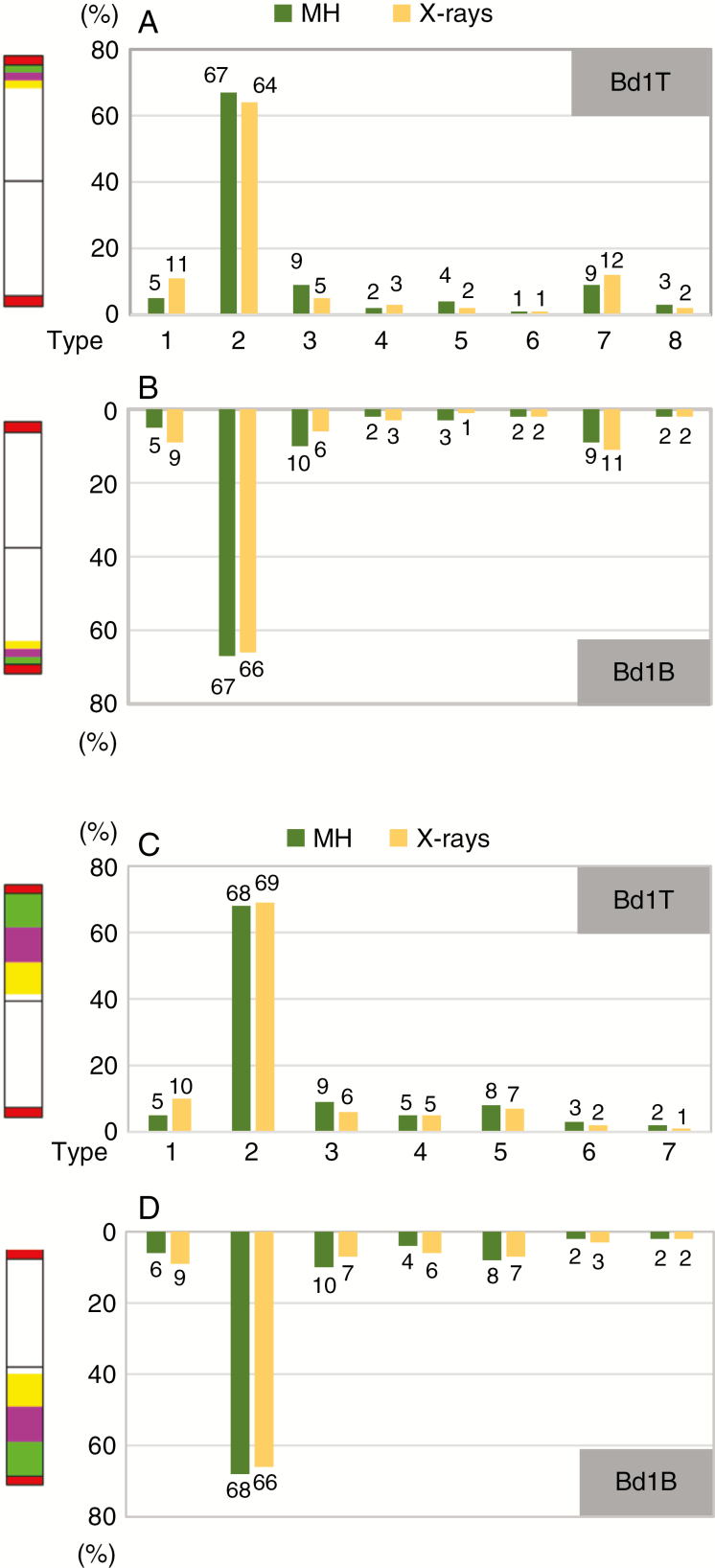
Frequencies of micronuclei with the signals of BAC pools that span the subterminal regions of Bd1T (A) and Bd1B (B) and of the telomeric probe as well as frequencies of micronuclei with the signals of BAC pools that span almost the entire Bd1T (C) and Bd1B (D) and of the telomeric probe in Brachypodium cells after MH and X-ray treatment. The datasets for two concentrations of MH and two doses of X-rays were combined.

The mcFISH approach, which combines Bd1-specific pools of BAC clones with a telomeric probe, permits the frequencies of specific micronucleus types to be assessed (Fig. 4). Because the frequencies of micronuclei with a different FISH signal composition did not depend on the doses of X-rays or the concentrations of MH that were used in this study, the data were merged into two datasets, respectively. Figure 4A shows the frequencies of micronucleus types that were related to the BAC pools that contained the distal region of Bd1T, while Fig. 4B is related to the corresponding region of Bd1B.

Statistical analyses using ANOVA and LSD multiple comparison tests reveal significant differences (*P* < 0.05) in the frequency of various micronuclei types. After both MH treatment and the X-rays, the frequency of micronucleus type 2 is significantly higher than the frequency of all other types (Table S3A, B). In contrast, after X-rays the frequency of type 3 micronuclei is significantly different from those of other types. However, in some cases statistically significant differences between the frequencies of particular micronuclei were not observed, as for types 1, 4, 5 and 8 after MH treatment, which were visualized in FISH with BACs spanning the subterminal region of Bd1T and with the telomeric probe (Table S3A). Similarly, frequencies of types 3, 4, 5 and 8 are not significantly different after X-rays and FISH with the BACs specific for the subterminal region of Bd1B and with the telomeric probe. There are no statistical differences between micronuclei types 1, 4, 5, 6 and 8 after MH treatment and between types 4, 5, 6 and 8 after X-rays (Table S3B).

Type 2 micronuclei (with one telomeric signal) are by far the most numerous, contributing 67 % of all of the micronuclei after MH treatment and 64 % and 66 % after X-ray treatment (Fig. 4A, B). Given that the Brachypodium genome consists of five chromosomes and that the BAC-based painting probes that we used are only diagnostic for the distal parts of chromosome Bd1, such a result is not unexpected. It is also consistent with our observations with repetitive DNA probes, and with sparse published data that confirm that distal chromosome regions are more prone to DSBs and involvement in micronuclei in both a small- (Brachypodium; Kus *et al.*, 2017) and a large-genome grass (barley; Juchimiuk *et al.*, 2007; Juchimiuk-Kwasniewska *et al.*, 2011). Interestingly, we show that MH is a stronger inducer of type 3 micronuclei, which consist of two telomeric signals (9 %, 10 %), than X-rays (5 %, 6 %; Fig. 4A, B). Similar frequencies of this type of MH-induced micronuclei were observed in FISH experiments using repetitive DNA sequences as probes (Kus *et al.*, 2017). FISH-negative micronuclei (type 1) were more frequent after X-rays (11 %, 9 %) than after MH treatment (5 %; Fig. 4A, B). These results suggest that X-rays lead to DNA breaks in interstitial chromosome regions at a higher frequency compared to MH.

Micronuclei with one telomeric signal and one signal from each labelled BAC pool (type 7) are the most frequent of this group. This type of micronucleus contributed to 9 % of the total after MH treatment and 12 % and 11 % after X-ray treatment (Fig. 4A, B). These results indicate that DSBs rarely occur within regions of Bd1 that are highlighted by the T- and B-pools, but rather that they take place in the FISH-negative parts. Breaks within the labelled BAC pools (micronuclei types 4, 5 and 6) were detected at consistently low frequencies after both MH treatment and X-rays (Fig. 4A, B). In summary, we conclude that the painting-positive distal regions of chromosome Bd1 are not ‘hot spots’ for mutations that are induced by MH and X-rays.

### Chromosome Bd1 is overrepresented in micronuclei

**Fig. 5.**
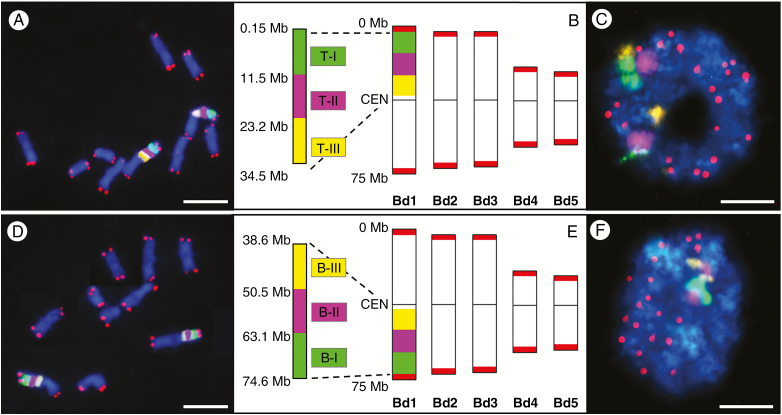
mcFISH with the large Bd1-specific BAC pools and the telomeric probe (red) in the control material. (A–C) The T-I (green), T-II (purple) and T-III (yellow) pools span almost the entire Bd1T. (D–F) The B-I (green), B-II (purple) and B-III (yellow) pools span almost the entire Bd1B. (A, D) Mitotic metaphase chromosomes. (B, E) Ideograms of the Brachypodium chromosomes with the approximate physical positions of the pools. CEN, centromere. (C, F) Interphase nuclei without micronuclei. Chromatin is stained with DAPI (blue). Scale bars = 5 μm.

To compare the structure and frequencies of micronuclei derived from distal chromosome fragments with those originating from much longer regions of chromosome arms, we used three (T-I, T-II, T-III) large pools of BACs that spanned 34.35 Mb along Bd1T (Fig. 5A, B), and three other pools (B-I, B-II, B-III) covering 36 Mb of Bd1B. These complex probes produced continuous linear signals along mitotic metaphase chromosome preparations, and occasionally overlapped slightly because of high chromatin condensation (Fig. 5A, D). However, they occupied specific and well-defined territories in interphase nuclei, together with clear and distinct signals of the telomeric probe (Fig. 5C, F).

After X-ray and MH treatment in this experiment, seven categories of micronuclei were revealed, designated types 1–7 (Fig. 3I, O). Again, because the same types of micronuclei were observed for both arms, only the nuclei with micronuclei that had hybridized with the telomeric probe and T-I, T-II and T-III pools are presented. The photomicrographs of micronuclei with the telomeric probe and BAC pools spanning almost entirely Bd1B are shown in Fig. S3. As the association of homologous chromosomes is common at interphase (Robaszkiewicz *et al.*, 2016), it precluded the effective counting of the signals within the original nuclei. Thus, we focused exclusively on the analysis of the signals in the micronuclei.

A type 1 FISH-negative micronucleus (Fig. 3I) is likely to be derived from an interstitial chromosome fragment of unknown origin, and therefore it is possible that Bd1B is also involved. The presence of one telomeric signal in a micronucleus (Fig. 3J; type 2) suggests its origin from a distal chromosome fragment of unknown origin, and again does not exclude the involvement of another arm of Bd1. A micronucleus with two telomeric signals (Fig. 3K; type 3) comprises two unrelated BAC-negative chromosome fragments or one laggard chromosome Bd2–Bd5. The micronucleus in Fig. 3L (type 4) contains two signals – one telomeric and one derived from BAC pool T-I. Such a composition implies that it was formed from a fragment of the top arm of Bd1 as the result of a DSB within T-I or between the T-I and T-II pools. The presence of one telomeric signal and two signals from the BAC clones, one of the T-I and one of the T-II BAC pool in micronucleus type 5, suggests that the DSB occurred either within the latter pool (Fig. 3M) or between the T-II and T-III pools. By contrast, the complex micronucleus presented in Fig. 3N (type 6) has four signals – one telomeric and three BAC probes, each of which was derived from a different pool. This indicates that the DNA break occurred either within the T-III BAC pool or was even more proximally localized within the DAPI-stained arm region of Bd1. We also observed the most structurally complex micronucleus type 7, which consists of two telomeric signals and three other signals, each from a different BAC pool, suggesting that it originated either from one complete, laggard Bd1 chromosome or from the entire Bd1T and a distal fragment that was derived from Bd1B or was of an unknown origin (Fig. 3O).

The application of mcFISH using telomeric and entire arm-painting Bd1-specific BAC probes in Brachypodium root-tip cells that had been treated with MH or X-rays permits the analysis of the relative frequencies of the different types of micronuclei (Fig. 4). Because the frequencies of micronuclei with different signal combinations did not depend on the concentration of MH or dose of X-rays, the data have been combined into two mutagen-specific datasets, as presented above.

ANOVA and LSD multiple comparison tests reveal again significant differences (*P* < 0.05) between the frequencies of the various types of micronuclei, which was the case of micronucleus type 2 (Table S3C, D). Interestingly, the frequency of X ray-induced type 5 micronuclei visualized by FISH with BACs spanning almost the entire Bd1T and with the telomeric probe (Table S3C), and the frequency of MH-induced type 3 micronuclei detected by BACs spanning Bd1B and with the telomeric probe (Table S3D) were shown to be significantly different from all other types. No statistically significant differences in frequencies are detected for some other micronuclei that have originated from Bd1T, for example MH-induced types 1, 4, 5, 6 and 7 and X ray-induced types 6 and 7 (Table S3C). Also, no significant differences were detected between the frequencies of Bd1B-originated micronuclei types 1, 4, 5, 6 and 7 after MH treatment and X ray-induced types 4, 5 and 6 (Table S3D).

The fact that type 2 micronuclei are the most frequent after both MH (68 %) and X-ray (69 %, 66 %; Fig. 4C, D) treatment implies that micronuclei are predominantly formed from a single, distal chromosome fragment regardless of the mutagen used. In contrast, micronuclei with two telomeric signals (type 3) are observed at a higher frequency after MH (9 %, 10 %) than after X-ray (6 %, 7 %; Fig. 4C, D) treatments. Conversely, the interstitial chromosome fragments that result from two DSBs seemed to be involved more frequently in the formation of micronuclei type 1 in the material that was subjected to X-radiation (10 %, 9 %) compared to MH (5 %, 6 %; Fig. 4C, D).

The discrimination of different regions of chromosome Bd1 enables the identification of the fragment of Bd1 in micronuclei, and allows us to compare the involvement of the top and bottom arms of this metacentric chromosome. The frequencies of micronuclei types 4–7, which contained different signals from all of the labelled T-I, T-II, T-III and B-I, B-II, B-III BAC pools, vary from 1 % to 8 % (Fig. 4C, D). The most abundant micronuclei in this group have one telomeric signal and two painting signals that were derived from the T-I and T-II pools (type 5). Other categories of micronuclei (types 4, 6 and 7) are observed at a relatively low frequency. This suggests that these micronuclei induced by MH and X-rays arise from large fragments rather than from small ones. Additionally, the type of mutagen used has no effect on significant differences in the composition of the micronuclei. Moreover, because the difference in the frequencies of the respective (1–7) types of micronuclei is not significant between Bd1T and Bd1B after MH and X-rays (Fig. 4C, D), these observations suggest that it is not specific DNA sequences but rather the position along the chromosome arms that is more crucial for inducing DSBs in Brachypodium after mutagens are applied.

The combined frequencies of micronuclei that had BAC signals (types 4–7) specific for Bd1T (Fig. 4C) and Bd1B (Fig. 4D) demonstrate that this chromosome is involved in micronucleus formation at a relatively high frequency after both MH treatment (34 %) and X-rays (33 %). Genome sequence information (IBI, 2010) and chromosome measurements (Lusinska *et al.*, 2018) show that Bd1 contributes to approx. 27 % of the Brachypodium genome. This, coupled with the observed frequencies of the type 4–7 micronuclei, suggests that the contribution of this chromosome to the formation of mutagen-induced micronuclei in Brachypodium root-tip cells is disproportionally higher than might be expected from random. Various aspects of chromatin organization, for example heterochromatin to euchromatin ratio and gene density, can influence the sensitivity to mutagens of different plant species and probably also specific regions in plant nuclear genomes (Juchimiuk *et al.*, 2006; Kwasniewska and Mikolajczyk, 2014). Information regarding the chromosomal distribution of these features of the Brachypodium genome is available (IBI, 2010). However, our analyses involved only one chromosome of the genome, which precludes an explanation of the overrepresentation of this chromosome in micronuclei.

Whilst our understanding of the origin of micronuclei in plants is limited, similar studies on human cells are relatively well advanced and some have shown a non-random involvement of specific chromosomes in micronuclei formation (Leach and Jackson-Cook, 2001; Chung *et al.*, 2002; Norppa and Falck, 2003). This phenomenon appears to be correlated with the specific agents that are used to induce micronuclei, as was reported by Chung *et al.* (2002), who demonstrated that 1,2,4-benzenetriol-induced micronuclei were formed from chromosome 8 most frequently. By contrast, chromosomes 1, 9, 15, 16 and Y were more frequently involved in micronuclei formation after 5-azacitidine treatment (Guttenbach and Schmid, 1994), while chromosomes 9 and 16 were overrepresented in mitomycin C-induced micronuclei (Hovhannisyan *et al.*, 2012). On the other hand, an apparently random origin of micronuclei was demonstrated in some other studies; for example, Wuttke *et al.* (1997) reported that chromosomes 2 and 7 form micronuclei at similar frequencies after X-ray treatment. In addition, ionizing radiation-induced micronuclei in lymphocytes have no significant preponderance of chromosomes 1, 7, 11, 14, 17 and 21 (Fimognari *et al.*, 1997). Although some studies have attempted to link the preponderance of specific chromosome in micronuclei with chromatin organization (Fauth *et al.*, 2000) and chromosome size and gene density (Puerto *et al.*, 1999), the mechanism of either the random or the non-random involvement of chromosomes remains unclear.

## CONCLUSION

Although knowledge about the composition of micronuclei in plants is still limited, BAC-FISH-based chromosome painting permits a detailed insight into their structure, allowing conclusions to be made about the mechanisms of their formation. It appears that the application of mutagenic treatments to Brachypodium, combined with its well-developed molecular and cytogenetic resources, makes this grass a very promising, if not unique, model system for studying mutagenesis amongst all monocotyledonous plants.

## SUPPLEMENTARY DATA

Supplementary data are available online at https://academic.oup.com/aob and consist of the following. Table S1: Characteristics of the BACs comprising the small pools used for specific painting of the subterminal regions of Brachypodium chromosome Bd1. Table S2 Characteristics of the BACs comprising the large pools used for specific painting of almost entire arms of Brachypodium chromosome Bd1. Table S3: Detailed statistical analyses related to the data presented in Fig. 4. Fig. S1: Ideogram of Brachypodium chromosomes showing the distribution of telomeric, centromeric, 5S rDNA and 35S rDNA sequences as determined by FISH. Fig. S2: Brachypodium interphase nuclei with micronuclei induced using MH treatment and X-radiation subjected to mcFISH with BAC pools spanning the subterminal region of Bd1B and with the telomeric probe. Fig. S3: Brachypodium interphase nuclei with micronuclei induced by MH treatment and X-radiation subjected to mcFISH with BAC pools spanning almost the entire Bd1B and with the telomeric probe.

Supplementary Table S1

Supplementary Table S2

Supplementary Table S3

Supplementary Figure S1

Supplementary Figure S2

Supplementary Figure S3
