# Supplementary material for: Dissecting the chromosomal composition of mutagen-induced micronuclei in Brachypodium distachyon using multicolour FISH
Source: Ann Bot. 2018 Jul 5;122(7):1161–71. doi: 10.1093/aob/mcy115 (PMC6324755; doi:10.1093/aob/mcy115)
Supplement: Supplementary Table S1 [file mcy115_suppl_supplementary_table_s1.pdf]

TABLE S1. Characteristics of the BACs comprising the small pools used for the specific painting of the subterminal regions of Brachypodium chromosome Bd1.

**Bd1T**				
Pool	Clone name	**Start (bp)**	**End (bp)**	**Repeat content (%)**
**T-I**	a0035K02	147863	304506	18.31
	b0027N17	560624	710332	6.56
	a0037D23	1171403	1328435	13.07
	a0012F06	1537097	1734409	7.59
	a0032E05	1907231	2063694	11.83
**T-II**	a0008O14	2635548	2801693	8.30
	a0021B03	3028832	3173186	6.23
	a0004B12	3276891	3460444	5.68
	b0044D24	3878248	4004060	13.91
	b0003A11	4404030	4546882	30.82
**T-III**	a0032K13	5048843	5206517	26.89
	a0017K22	5375697	5509098	17.71
	b0037O18	6122656	6272292	19.17
	a0022C04	6574012	6741405	30.61
	b0040G07	7221475	7389553	30.09

**Bd1B**				
Pool	Clone name	**Start (bp)**	**End (bp)**	**Repeat content (%)**
**B-III**	a0019B19	67392232	67529032	8.00
	b0003K24	67945518	68072820	13.86
	a0011O07	68533765	68686250	24.11
	a0043A05	68898017	69053532	12.54
	b0004O01	69023274	69164463	23.47
**B-II**	b0039M08	69966292	70146601	29.34
	a0040G14	70435911	70578835	17.94
	b0017K19	71146553	71281318	0.00
	a0021F18	71455475	71597258	0.00
	a0041A08	72027767	72181888	6.97
**B-I**	b0002N07	72465040	72619352	4.45
	a0005K09	72948475	73083942	4.87
	b0039K17	73601518	73740071	5.12
	a0033F06	74020535	74180685	4.77
	b0035K23	74475472	74659792	12.79
