# Supplementary material for: Dissecting the chromosomal composition of mutagen-induced micronuclei in Brachypodium distachyon using multicolour FISH
Source: Ann Bot. 2018 Jul 5;122(7):1161–71. doi: 10.1093/aob/mcy115 (PMC6324755; doi:10.1093/aob/mcy115)
Supplement: Supplementary Table S2 [file mcy115_suppl_supplementary_table_s2.pdf]

TABLE S2. Characteristics of the BACs comprising the large pools used for the specific painting of almost entire arms of Brachypodium chromosome Bd1.

**Bd1T**				
**Pool**	**Clone identifier**	**Start (bp)**	**End (bp)**	**Repeat content (%)**
T-I	a0035K02	147863	304506	18.31
	b0027N17	560624	710332	6.56
	a0037D23	1171403	1328435	13.07
	a0012F06	1537097	1734409	7.59
	a0032E05	1907231	2063694	11.83
	a0008O14	2635548	2801693	8.30
	a0021B03	3028832	3173186	6.23
	a0004B12	3276891	3460444	5.68
	b0044D24	3878248	4004060	13.91
	b0003A11	4404030	4546882	30.82
	a0032K13	5048843	5206517	26.89
	a0017K22	5375697	5509098	17.71
	b0037O18	6122656	6272292	19.17
	a0022C04	6574012	6741405	30.61
	b0040G07	7221475	7389553	30.09
	b0013P15	7656602	7805960	29.21
	a0013O16	7926543	8055682	39.20
	b0030L10	8680898	8845282	10.03
	b0012L20	8850673	9007358	14.08
	a0015H06	9424591	9569999	14.80
	a0007G23	9965773	10101237	15.91
	a0032D10	10490542	10652198	11.18
	a0003J21	10927667	11073109	16.93
T-II	a0023P13	11505702	11632287	22.48
	a0032F08	12115980	12241228	17.12
	a0024M10	12444261	12575731	23.81
	a0027D04	12706461	12847057	22.87
	b0019G20	13362834	13517753	21.02
	b0023C02	13999817	14137163	13.45
	b0001G04	14561692	14709543	15.53
	a0020A04	15092918	15238493	0.00
	b0023O18	15449374	15577903	0.00
	a0003N21	16107590	16251236	10.80
	a0002N01	16344468	16496573	11.12
	a0009N18	17150298	17335777	0.00
	a0014L23	17404191	17543242	7.86
	b0018P22	18190466	18326563	23.15
	a0017E13	18574112	18708723	0.00
	a0010I03	19198770	19342731	7.06
	a0007L04	19364297	19511432	8.67

Bd1T				
**Pool**	**Clone identifier**	**Start (bp)**	**End (bp)**	**Repeat content (%)**
**T-II (cont.)**	b0002O16	20013520	20160236	0.00
	a0024N14	20488400	20631457	19.18
	a0027K03	21168673	21307307	0.00
	a0010K04	21496092	21643627	19.83
	a0011I01	21907910	22040598	0.00
	a0018B03	22412015	22565632	32.71
	b0022H13	23114454	23242441	0.00
**T-III**	a0023E14	23230575	23392276	21.52
	a0043B06	24028749	24191469	22.76
	a0042C21	24228323	24375228	16.48
	a0026E19	25017625	25161139	14.31
	a0046B12	25556278	25718695	17.60
	a0018O15	25727688	25878318	13.18
	b0028A06	26442023	26591576	16.02
	b0002C04	27060765	27214938	27.84
	a0006K13	27522409	27682274	23.09
	a0044I06	28135872	28292480	21.81
	a0043P17	28526824	28683718	21.98
	a0029A09	28940084	29079192	22.18
	a0032C01	29475135	29677346	31.42
	a0002G12	30075959	30187436	25.08
	a0007B20	30824142	30835981	8.00
	a0036J15	31222238	31387974	13.86
	a0037D16	31313973	31498115	24.11
	a0018G20	32094917	32274028	12.54
	b0024I19	32507293	32633286	0.00
	b0014O02	33010624	33123772	0.00
	a0024G16	33587038	33745554	0.00
	b0037O03	33832455	34023561	20.87
	a0004L01	34316249	34466638	15.53

Bd1B				
**Pool**	**Clone identifier**	**Start (bp)**	**End (bp)**	**Repeat content (%)**
**B-III**	b0011C11	38625084	38768816	25.98
	a0002G19	39219901	39352626	20.11
	a0030K01	39352642	39424325	13.60
	a0002I22	39952805	40102980	19.01
	b0023K21	40363127	40508554	24.17
	a0003G01	41070484	41199669	5.68
	a0017I18	41400831	41536057	10.35
	a0006H08	42291978	42445395	30.82
	a0034B17	42516220	42665540	26.89
	a0022M24	43211660	43355407	17.71

Bd1B				
**Pool**	**Clone identifier**	**Start (bp)**	**End (bp)**	**Repeat content (%)**
**B-III (cont.)**	b0004P09	43536825	43670757	0.00
	b0003O14	43968448	44100412	0.00
	a0046B04	44701450	44835069	28.04
	a0009H21	45130478	45275483	30.09
	b0019N18	45624834	45763826	29.21
	b0039A23	45904934	46085862	34.61
	b0025P22	46564769	46692954	10.03
	a0045K11	47017729	47159607	14.08
	a0018I01	47327837	47472575	14.80
	a0018A03	48153393	48351371	12.13
	b0044L08	48612347	48783561	9.01
	a0016L07	49131764	49282548	17.24
	b0042L08	49567141	49756854	22.48
	a0003G14	50139085	50274374	17.12
**B-II**	a0007A17	50488742	50629310	23.81
	a0002G03	50987420	51131768	21.02
	a0002M19	51404954	51599184	13.45
	b0035K24	51720482	51914140	15.53
	a0046C24	52577876	52717406	12.29
	a0020C13	52998818	53110321	14.83
	a0011F10	53395079	53532078	10.55
	a0046G17	54082210	54253048	0.00
	b0047M09	54775761	54934862	11.12
	b0036M23	55099293	55274619	14.70
	a0011D03	55361479	55401129	7.86
	b0044C20	56154348	56285508	16.71
	b0013C18	56402015	56523891	0.00
	b0028P17	57093738	57225377	0.00
	a0022N20	57208701	57348499	11.96
	a0019B04	58011832	58155264	19.18
	b0003K21	58350464	58480351	15.82
	a0010A14	59503419	59676696	0.00
	a0008E12	60079495	60251020	17.16
	a0005H16	60258365	60413281	0.00
	b0037A14	61095306	61288697	19.17
	a0012H18	61475648	61619144	0.00
	b0003A21	61920716	62077069	0.00
	a0034M17	62501248	62642447	0.00
**B-I**	a0045D19	63062019	63221983	14.31
	b0022G04	63557791	63711230	17.60
	a0013D23	64120769	64297730	16.02
	b0011I02	64559074	64702171	27.84
	a0003I14	65067565	65202176	23.09
	a0046P14	65376014	65522455	21.81
	a0009I15	65946210	66098108	21.98

Bd1B				
**Pool**	**Clone identifier**	**Start (bp)**	**End (bp)**	**Repeat content (%)**
**B-I (cont.)**	b0026H13	66197674	66346594	22.18
	b0030D22	67065313	67205367	25.08
	a0019B19	67392232	67529032	8.00
	b0003K24	67945518	68072820	13.86
	a0011O07	68533765	68686250	24.11
	a0043A05	68898017	69053532	12.54
	b0004O01	69023274	69164463	23.47
	b0039M08	69966292	70146601	29.34
	a0040G14	70435911	70578835	17.94
	b0017K19	71146553	71281318	0.00
	a0021F18	71455475	71597258	0.00
	a0041A08	72027767	72181888	6.97
	b0002N07	72465040	72619352	4.45
	a0005K09	72948475	73083942	4.87
	b0039K17	73601518	73740071	5.12
	a0033F06	74020535	74180685	4.77
	b0035K23	74475472	74659792	12.79
