# Supplementary material for: Dissecting the chromosomal composition of mutagen-induced micronuclei in Brachypodium distachyon using multicolour FISH
Source: Ann Bot. 2018 Jul 5;122(7):1161–71. doi: 10.1093/aob/mcy115 (PMC6324755; doi:10.1093/aob/mcy115)
Supplement: Supplementary Table S3 [file mcy115_suppl_supplementary_table_s3.pdf]

TABLE S3. Detailed statistical analyses related to the data presented in Fig. 4. Values are means ±standard error (n = 3). Means followed by the same letter (a, b, c, d) are not significantly different from each other using the parametric analysis of variance and *post hoc* LSD test ($p <$ 0.05).

A.

MH Type 1	4.00±0.58 c
MH Type 2	54.00±1.53 a
MH Type 3	7.33±0.88 b
MH Type 4	1.67±0.33 cd
MH Type 5	3.33±0.88 c
MH Type 6	0.67±0.33 d
MH Type 7	7.33±0.33 b
MH Type 8	2.33±0.33 cd

X-ray Type 1	9.33±0.88 c
X-ray Type 2	54.33±1.45 a
X-ray Type 3	4.33±0.88 b
X-ray Type 4	2.67±0.33 bd
X-ray Type 5	1.67±0.33 bd
X-ray Type 6	1.00±0.57 d
X-ray Type 7	10.33±1.20 c
X-ray Type 8	1.67±0.88 bd

B.

MH Type 1	4.00±0.58 c
MH Type 2	53.00±2.31 a
MH Type 3	8.00±0.58 b
MH Type 4	1.67±0.88 c
MH Type 5	2.33±0.66 c
MH Type 6	1.67±0.33 c
MH Type 7	7.00±0.58 b
MH Type 8	1.67±0.33 c

X-ray Type 1	7.33±0.67 c
X-ray Type 2	54.67±0.88 a
X-ray Type 3	5.00±0.58 b
X-ray Type 4	2.33±0.67 d
X-ray Type 5	0.67±0.33 d
X-ray Type 6	1.53±0.88 d
X-ray Type 7	9.00±0.58 c
X-ray Type 8	1.67±0.33 d

C.

MH Type 1	4.00±0.58 bc
MH Type 2	52.00±2.19 a
MH Type 3	7.00±0.58 b
MH Type 4	4.00±0.58 bc
MH Type 5	4.00±1.00 bc
MH Type 6	2.33±0.33 c
MH Type 7	1.67±0.33 c

X-ray Type 1	8.00±0.58 c
X-ray Type 2	55.00±1.15 a
X-ray Type 2	6.00±0.58 b
X-ray Type 4	8.00±0.58 c
X-ray Type 5	4.00±0.58 d
X-ray Type 6	1.67±0.33 e
X-ray Type 7	0.67±0.33 e

D.

MH Type 1	4.67±0.33 c
MH Type 2	54.00±1.73 a
MH Type 3	8.00±0.58 b
MH Type 4	4.67±0.33 c
MH Type 5	3.00±1.00 c
MH Type 6	1.67±0.33 c
MH Type 7	1.67±0.88 c

X-ray Type 1	8.00±1.15 b
X-ray Type 2	53.00±1.73 a
X-ray Type 3	5.67±0.88 bc
X-ray Type 4	8.00±1.15 b
X-ray Type 5	4.67±0.33 c
X-ray Type 6	2.33±0.33 cd
X-ray Type 7	1.67±0.33 d
