# Supplementary figures and images for: Dissecting the chromosomal composition of mutagen-induced micronuclei in Brachypodium distachyon using multicolour FISH
Source: Ann Bot. 2018 Jul 5;122(7):1161–71. doi: 10.1093/aob/mcy115 (PMC6324755; doi:10.1093/aob/mcy115)

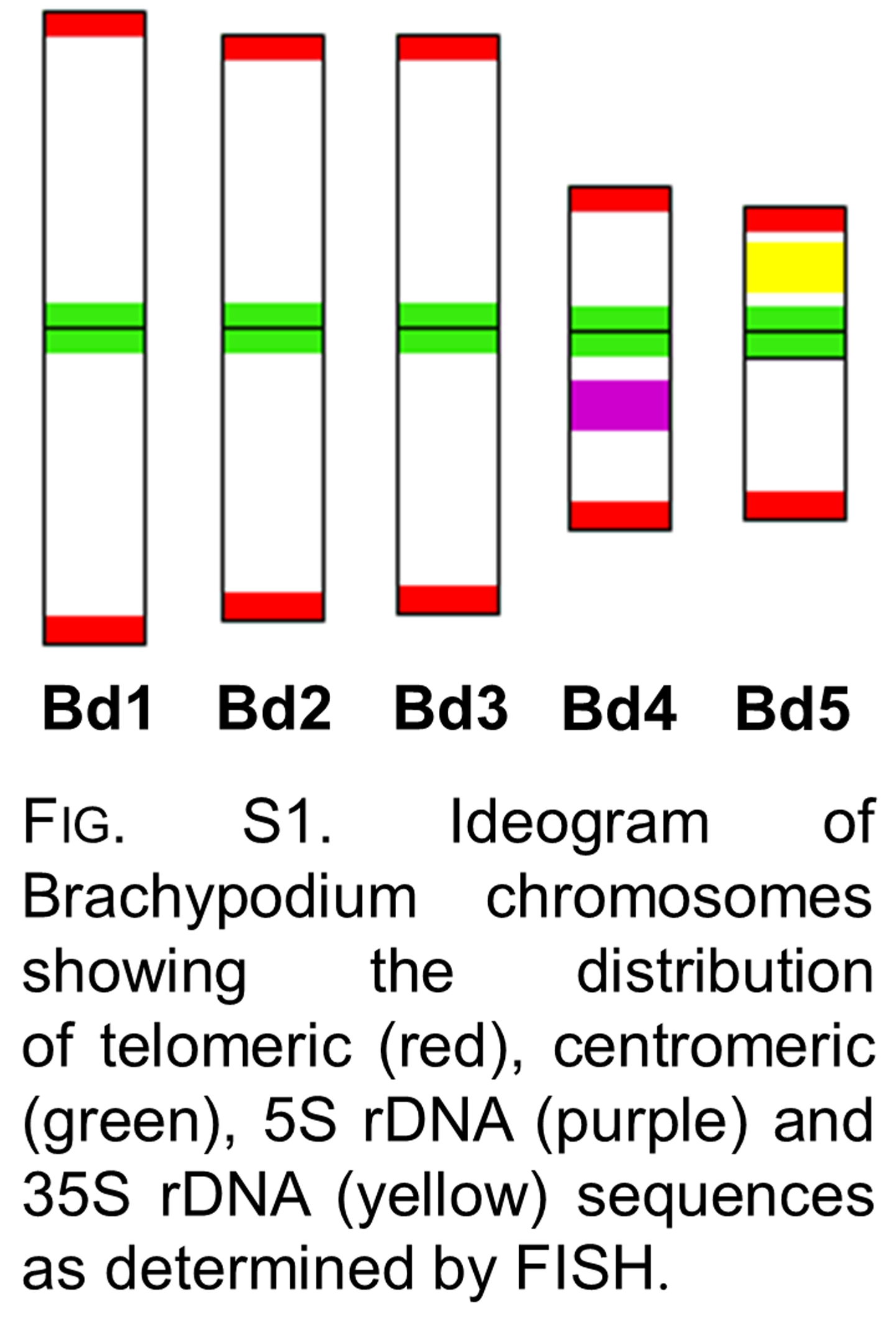

Supplement: Supplementary Figure S1 [file mcy115_suppl_supplementary_figure_s1.png]

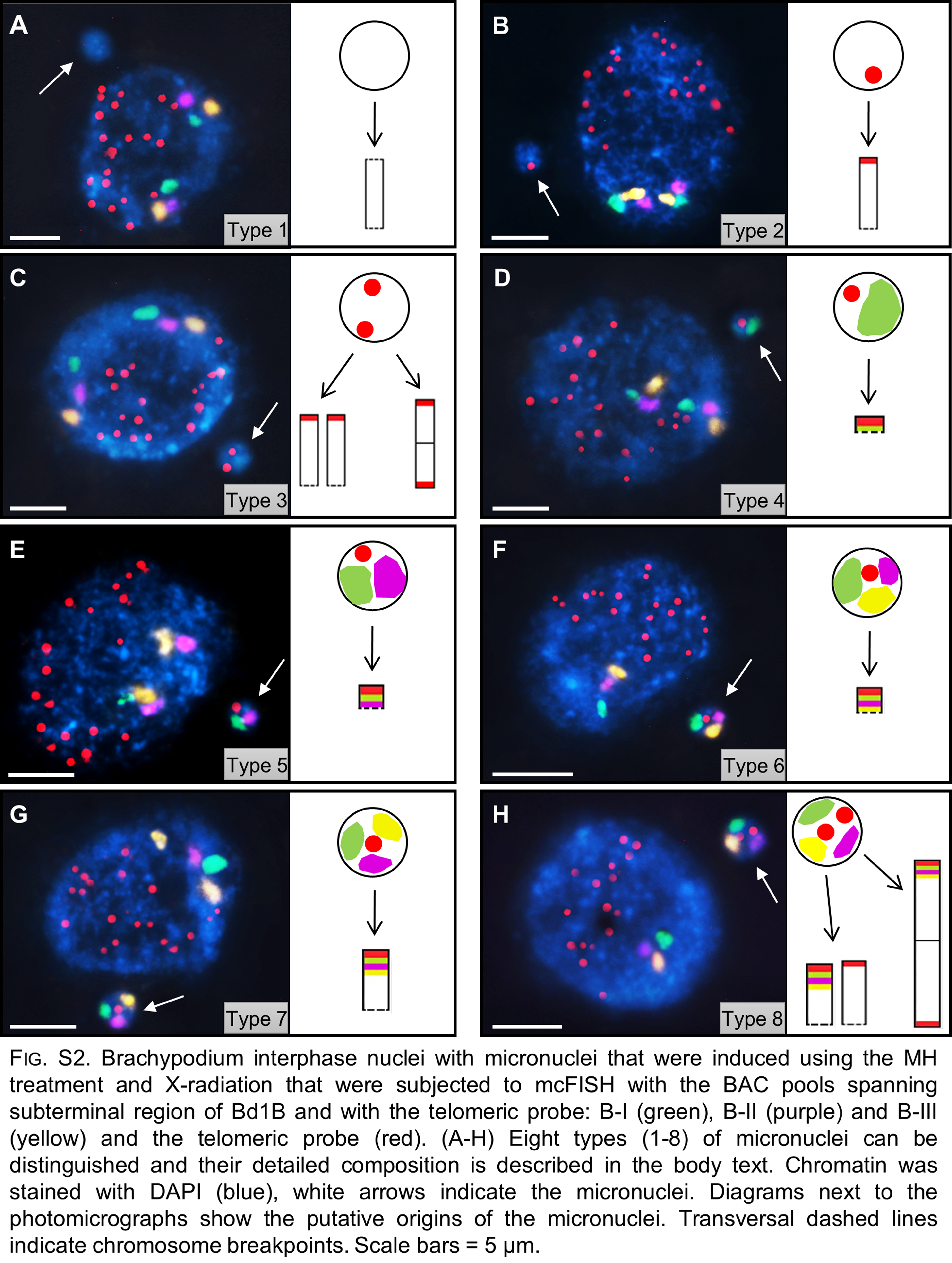

Supplement: Supplementary Figure S2 [file mcy115_suppl_supplementary_figure_s2.png]

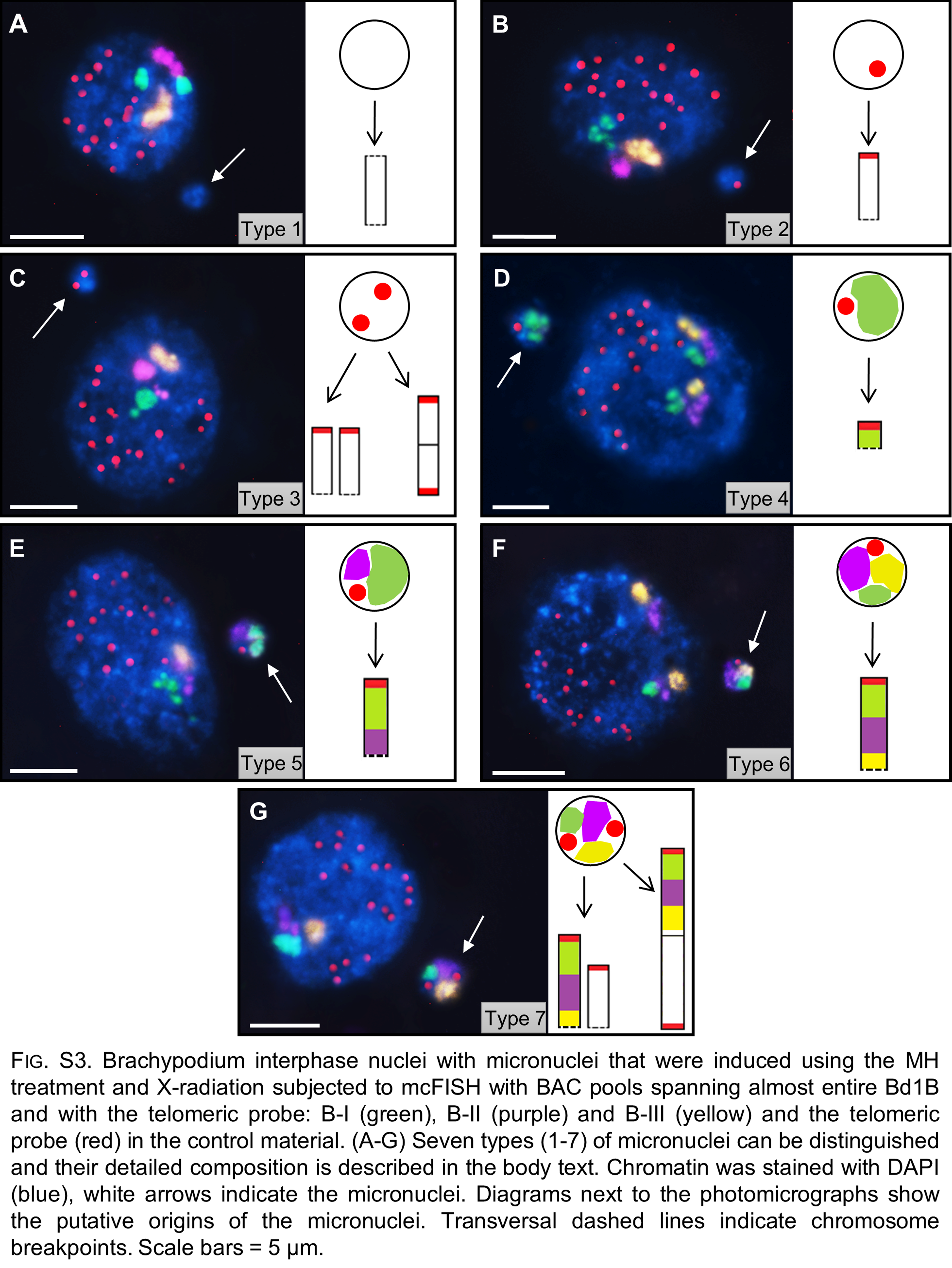

Supplement: Supplementary Figure S3 [file mcy115_suppl_supplementary_figure_s3.png]
